# Human Microglial Cells Synthesize Albumin in Brain

**DOI:** 10.1371/journal.pone.0002829

**Published:** 2008-07-30

**Authors:** Sung-Min Ahn, Kyunghee Byun, Kun Cho, Jin Young Kim, Jong Shin Yoo, Deokhoon Kim, Sun Ha Paek, Seung U. Kim, Richard J. Simpson, Bonghee Lee

**Affiliations:** 1 Center for Genomics and Proteomics, Lee Gil Ya Cancer and Diabetes Institute, Gachon University of Medicine and Science, Incheon, Korea; 2 Mass Spectrometry Analysis Group, Korea Basic Science Institute, Daejeon, Korea; 3 Department of Neurosurgery, Clinical Research Institute, Seoul National University Hospital, Seoul National University College of Medicine, Seoul, Korea; 4 Gachon Institute for Regenerative Medicine, Gachon University of Medicine and Science, Incheon, Korea; 5 Joint ProteomicS Laboratory, Ludwig Institute for Cancer Research & the Walter and Eliza Hall Institute of Medical Research, Melbourne, Australia; 6 Department of Medicine, University of British Columbia, Vancouver, Canada; Deutsches Krebsforschungszentrum, Germany

## Abstract

Albumin, an abundant plasma protein with multifunctional properties, is mainly synthesized in the liver. Albumin has been implicated in Alzheimer's disease (AD) since it can bind to and transport amyloid beta (Aβ), the causative agent of AD; albumin is also a potent inhibitor of Aβ polymerization. Despite evidence of non-hepatic transcription of albumin in many tissues including kidney and pancreas, non-hepatic synthesis of albumin at the protein level has been rarely confirmed. In a pilot phase study of Human Brain Proteome Project, we found evidence that microglial cells in brain may synthesize albumin. Here we report, for the first time, the *de novo* synthesis of albumin in human microglial cells in brain. Furtherore, we demonstrate that the synthesis and secretion of albumin from microglial cells is enhanced upon microgial activation by Aβ_1–42_- or lipopolysaccharide (LPS)-treatment. These data indicate that microglial cells may play a beneficial role in AD by secreting albumin that not only inhibits Aβ polymerization but also increases its clearance.

## Introduction

Albumin is the most abundant plasma protein with multifunctional properties such as ligand-binding and transport, maintaining the colloid osmotic pressure of plasma, and regulating neutrophil function [1]. Clinically, albumin has been extensively used in critical conditions including vascular collapse in severely ill patients and cirrhosis [2]. Recently, it has been also suggested that albumin specifically bind to low molecular weight molecules that might be important diagnostic or prognostic indicators of diseases [3].

Albumin is not only a high-abundance protein in plasma, but also a major component of most extracellular fluids including interstitial fluid, lymph, and cerebrospinal fluid (CSF) [4]–[7] since it enters tissues and organs from blood. For example, albumin enters brain across blood-brain barrier by molecular diffusion [8]. Albumin is found at a low concentration (∼0.2 g/L) in CSF, yet it amounts to ∼80% of the total CSF protein in contrast to ∼60% as in plasma [1]. CSF serum quotient of albumin, along with other blood-derived proteins in CSF, is widely used in the diagnosis of neurological diseases [9]. Albumin has been implicated in Alzheimer's disease (AD) because it can specifically bind to and transport amyloid beta (Aβ), the causative agent of AD [10], under physiological conditions [11]. Moreover, albumin is a potent inhibitor of Aβ polymerization, representing ∼60% of amyloid inhibitory activity in CSF and plasma [12].

Albumin is mainly synthesized in the liver at a rate of ∼12 g per day, representing ∼25% of total hepatic protein synthesis [13]. Non-hepatic transcription of albumin has been reported in kidney and pancreas [14], as well as in intestine, lymph gland, testicle, uterus, tongue, and mammary gland [15]. Despite the evidence of non-hepatic transcription of albumin in many tissues, non-hepatic synthesis of albumin at the protein level has been rarely confirmed. For example, albumin in thyroid gland was found to originate from blood rather than by *de novo* synthesis [16]. Also, cell-bound albumin, present on the surface of lymphocytes and macrophages, was found to originate either from the tissue culture medium in vitro or from serum in vivo [17]. Recently, Yamaguchi *et al*. [18] provided convincing evidence of albumin synthesis in bone tissues cultured in serum-, albumin-free medium. There has been no specific report about the synthesis of albumin in brain either at the mRNA level or at the protein level.

In a pilot phase study of Human Brain Proteome Project, we found evidence that microglial cells in brain may synthesize albumin. To further confirm this finding, we examined the expression of albumin in human microglial cells and brain tissues.

## Results and Discussion

To provide evidence of albumin synthesis in human microglial cells, three biospecimens were used: 1) immortalized human microglial cell line (HMO6); 2) human primary microglial cells; 3) human fetal and adult brain tissues. Additionally, Alzheimer's brain tissues were used to show the increased synthesis of albumin in microglial cells in AD.

**Figure 1:**
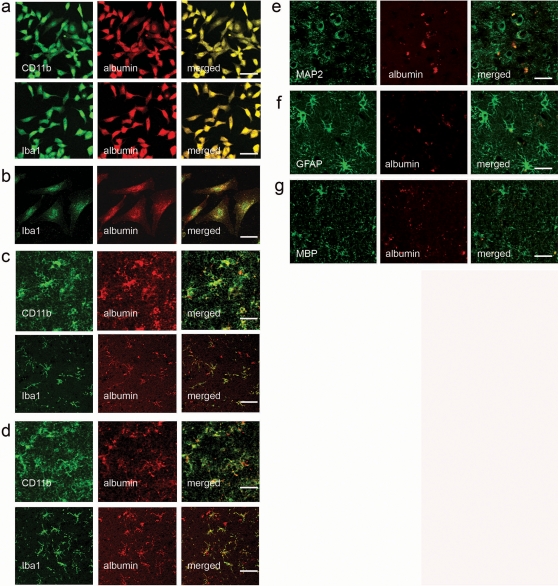
Albumin expression in human microglial cells. Microglial markers (CD11b or Iba-1) were co-expressed with albumin in all HMO6 cells (a) and human primary microglial cells (b). This observation was further confirmed by immunohistochemical staining of human fetal (c) and adult (d) brain tissues, in which albumin was co-expressed with CD11b or Iba-1. Anti-human-albumin antibody used for ICC and IHC does not have cross-reactivity with bovine albumin. Minimal co-localization of albumin and non-microglial markers was observed in other cell types of brain: MAP2 for neurons (e); GFAP for astrocytes (f); MBP for oligodendrocytes (g). Scale bars = 50 µm.

We used immunostaining for albumin and microglial markers in human cells and brain tissues to confirm that albumin is expressed in microglial cells both in vitro and in vivo. In immunocytochemistry (ICC), all HMO6 cells were double-positive for microglial markers (CD11b [19] and Iba1 [20]) and albumin (Fig. 1a); human primary microglial cells staining positive for Iba1 coexpressed albumin (Fig. 1b). In immunohistochemistry (IHC) study using human fetal and adult brain tissues, cells staining positive for microglial markers (CD11b and Iba1), also coexpressed albumin (Fig. 1c&d). When human adult brain tissues were double stained for albumin and non-microglial cell markers (i.e. microtubule-associated protein 2 (MAP2) for neurons; glial fibrillary acidic protein (GFAP) for astrocytes; myelin basic protein (MBP) for oligodendrocytes), minimal co-localization was observed (Fig. 1e–g).

**Figure 2:**
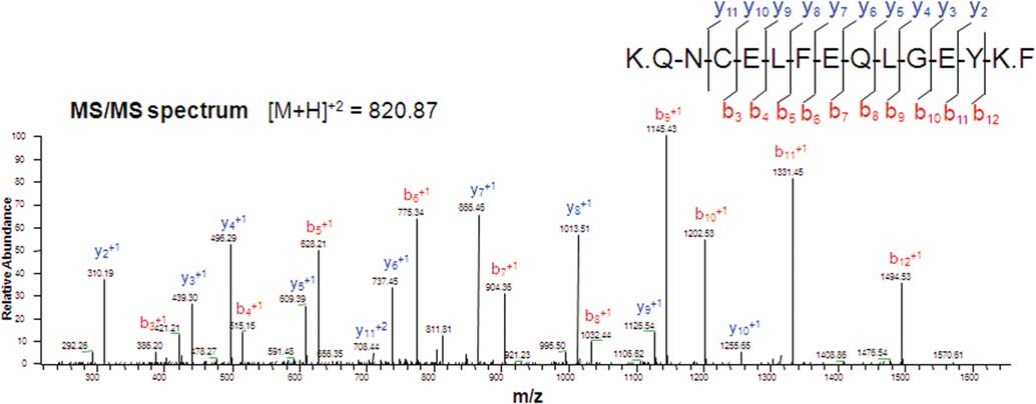
Representative MS/MS spectra of a tryptic peptide of albumin. Peptide sequencing provided clear evidence that albumin found in HMO6 cells is not bovine but human origin. The peptide QNCELFEQLGEYK is human-specific (i.e. the sequence is not 100% homologous with its bovine homologue, and thus distinguishable using MS/MS. The list of tryptic peptides identified using MS/MS is summarized in Table S1. b and y represent b ions and y ions respectively, which are generated during peptide fragmentation in MS/MS [29].

To further confirm that albumin detected in HMO6 cells is not bovine albumin from the incubation media but human albumin, peptide sequencing using tandem mass spectrometry (MS/MS) was performed, which provided clear evidence that the albumin in HMO6 cells is human origin (Fig. 2). The list of tryptic peptides identified using MS/MS is summarized in Table S1.

**Figure 3:**
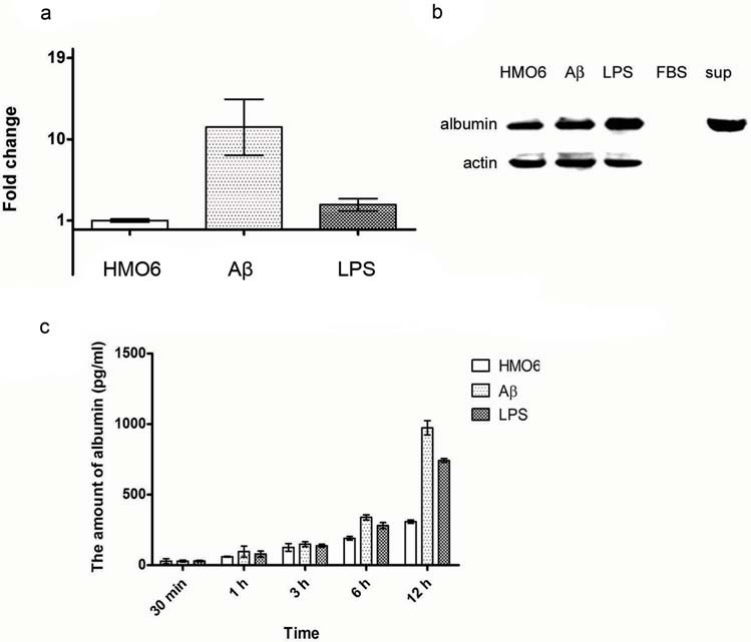
Increase in albumin synthesis and secretion after microglial activation by Aβ_1–42_- or LPS-treatments. qRT-PCR data (a) show that the transcription of albumin gene is significantly enhanced after microglial activation by Aβ_1–42_- or LPS-treatment. Immunoblot data also show that albumin synthesis increases at the protein level after microglial activation (b). In addition, immunoblot data show that anti-human-albumin antibody used does not have any cross-reactivity with bovine albumin, and that albumin is present in the incubation medium (i.e. thus secreted from cells). ELISA data show that albumin secretion from HMO6 cells increased significantly after microglial activation (c). Moreover, the level of albumin in the incubation medium of Aβ_1–42_-treated cells was significantly higher than that of LPS-treated cells. This observation partly explains why the increase of albumin expression at the mRNA level is not reflected well at the protein level. Since albumin is directly secreted from cells after synthesis, the increase of albumin expression seems to be reflected better in the incubation media than in cell lysates.

HMO6 cells were activated by either Aβ_1–42_ or lipopolysaccharide (LPS) as previously described [21]. Interestingly, the levels of albumin mRNA and protein increased upon microglial activation by Aβ_1–42_- or LPS- treatments (Fig. 3). According to quantitative real-time PCR results, albumin gene transcription in HMO6 cells was more responsive to Aβ_1–42_ than to LPS (Fig. 3a), which did not correlate well with albumin protein synthesis as illustrated by immunoblot analysis using HMO6 cell lysates (Fig. 3b). This may be partly explained by the fact that albumin is directly secreted from cells after synthesis. In accordance with this explanation, the level of albumin secreted by HMO6 cells into the incubation media also increased significantly after microglial activation, and was more responsive to Aβ_1–42_ than LPS (Fig. 3c).

The synthesis of albumin by microglial cells provides a convincing explanation why the proportion of albumin in CSF is higher than that in plasma. Since all blood proteins traverse blood-brain barrier by passive diffusion, larger molecules such as albumin are slower in exchange according to the laws of diffusion, and thus the relative proportion of albumin in CSF should be lower than that in plasma [22]. On the contrary, the proportion of albumin in CSF (∼80%) is higher than that in plasma (∼60%), which may be explained by *de novo* synthesis of albumin in brain by microglial cells. The important and diverse roles of albumin in extracellular fluids underscore the roles of microglial cells in maintaining microenvironments of brain under physiological conditions.

**Figure 4:**
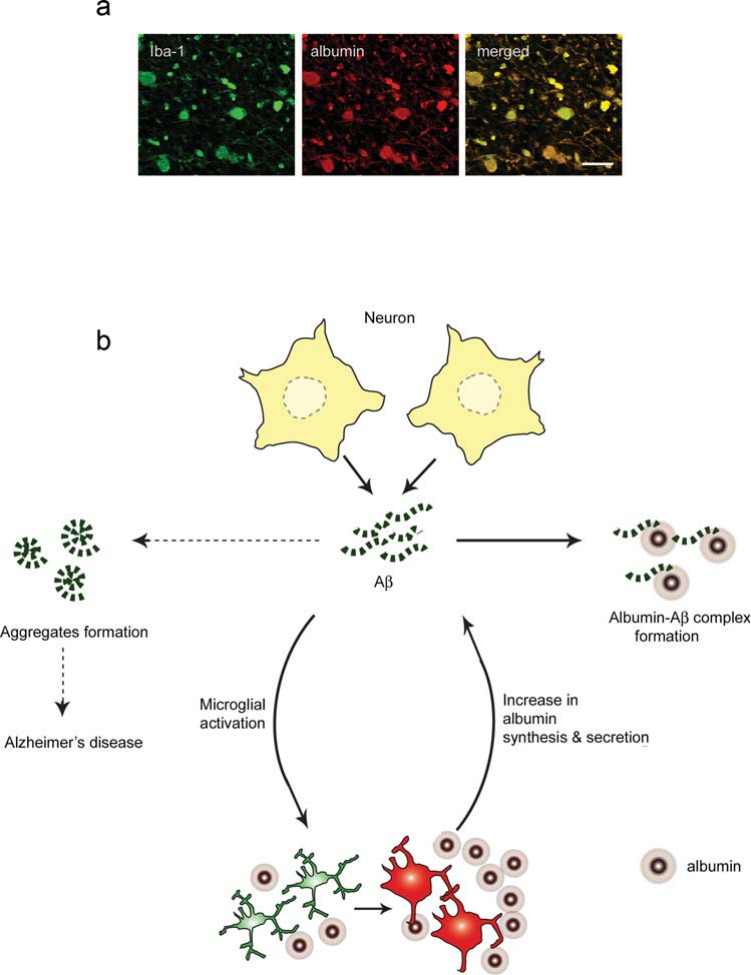
A proposed role of albumin secreted from human microglial cells in brain. a. Microglial cells synthesize albumin not only in physiological condition, but also in pathological condition at an increased level. Images shown were taken from Alzheimer's brain tissues. Iba1, a microglial marker, and albumin were co-localized. b. Microglial cells synthesize albumin in a physiological condition. When activated (e.g., by Aβ_1–42_), microglial cells surround amyloid deposition, recruit/activate more microglial cells, and increase albumin synthesis and secretion. Albumin secreted into tissue interstitial fluid inhibits Aβ polymerization and increase its clearance.

Our data also show that the expression level of albumin in microglial cells increases upon activation with Aβ_1–42_- or LPS- treatments. Microglial cells have been implicated in AD mainly because of their markedly elevated distribution in brain regions with Aβ deposition, and their pro-inflammatory functions [23]. Despite all the circumstantial evidence against microglial involvement in AD, it is still not clear whether microglial cells are ‘friends or foes’ in AD [24]. Our findings suggest that microglial cells may diminish Aβ deposition by increasing albumin synthesis and secretion. The capacity to bind to and transport Aβ enables albumin to inhibit Aβ polymerization and to increase Aβ clearance [12], [25] (Fig. 4). These findings indicate that microglial cells may play a beneficial role in AD in which the ‘amyloid hypothesis’ that Aβ aggregates to trigger a complex pathologic cascade leading to neurodegeneration is generally accepted [26].

## Materials and Methods

### Cell culture

For *in vitro* study, immortalized human microglial cell line (HMO6) was used [21]. HMO6 cells were grown in Dulbecco's modified Eagle's medium (DMEM, Gibco) with high glucose supplemented with 10% fetal bovine serum (FBS, Gibco) and 20 µg/ml gentamicin (Sigma), and incubated at 37°C and 5% CO_2_. LPS and Aβ_1–42_ were acquired from Sigma-Aldrich and added to HMO6 cells at a concentration of 100 ng/mL and 5 mM, respectively. Either LPS- or Aβ_1–42_-treated HMO6 cells were harvested 6 hrs after treatment for further analysis except ELISA in which the incubation media were collected along 12 hrs time course for analysis.

### Primary human microglial cell culture

Primary human microglial cells were prepared from embryonic human brains of 12–15 weeks gestation as previously described [21]. The use of embryonic tissue samples were approved by Ethics Committee of the University of British Columbia, Faculty of Medicine. Briefly, brain tissues were incubated in phosphate-buffered saline (PBS) containing 0.25% trypsin and 40 µg/ml DNase I for 30 min at 37°C. Dissociated cells were suspended in DMEM supplemented with 5% FBS, 5% horse serum, 20 µg/ml gentamicin, and 2.5 µg/ml amphotericine B (feeding medium), plated at a density of 10^6^ cells/ml in 10 cm culture dish (10 ml), and incubated at 37°C in an incubator with 5% CO_2_/95% air atmosphere. After 2–3 weeks *in vitro*, microglia-enriched cultures were prepared by harvesting the floating cells in culture dish and replating them on Lab-Tek II Chamber Slide System (210^4^ cells/wells, Nunc) for immunocytochemistry.

### Immunoblot

Cell lysates were prepared with lysis buffer containing 7M urea, 2M thiourea, and 4% CHAPS. 2 ml of the incubation medium was harvested, centrifuged at 1500 rpm for 10 minutes, and the supernatant was concentrated to about 15 µl using an Amicon centrifugal filter with 10-kDa nominal molecular weight limit (Millipore). Cell lysates, the incubation medium concentrate, and FBS were separated in 4–12% polyacrylamide gels (Invitrogen) and transferred to nitrocellulose membrane. The primary antibodies used were anti-human-albumin (1∶1000, Abcam) that does not have cross-reactivity with bovine albumin, and anti-β-actin (1∶1000, Cell Signaling).

### Quantitative real-time PCR (qRT-PCR)

RNA was isolated from six biological replicates from each group using Qiagen RNeasy MiniKit (Qiagen), pooled, and subjected to first-strand cDNA synthesis using Reverse Transcription System (Promega) according to the manufacturer's protocol. qRT-PCR was performed using Rotor-Gene 6000 (Corbett Lifescience), threshold cycle number and reaction efficiency were determined using Rotor-Gene 6000 series software version 2.7, and the 2^−ΔΔC^T method was used for relative quantitation. The primers used were: 5′-ATGCCCCGGAACTCCTTTTC -3′ (forward) and 5′-CAACAGGCAGGCAGCTTTAT -3′ (reverse) for albumin, and 5′-CTAGAAGCATTTGCGGTGGACGATGGAGGG -3′ (forward) and 5′-TGACGGGGTCACCCACACACTGTGCCCATCTA -3′ (reverse) for GAPDH.

### Enzyme-Linked Immunosorbent Assay (ELISA)

The amount of albumin in the cell incubation media was determined by human albumin BioAssay ELISA kit (US Biological). Six biological replicates were used, and each replicate was measured in duplicate.

### Immunocytochemistry (ICC)

Cells were grown on Lab-Tek II chamber slide (Nunc), rinsed in PBS, fixed in 4% paraformaldehyde for 20 min, and rinsed again in PBS. The cells were incubated for overnight at 4°C with mouse anti-human-albumin antibody (1∶200, R&D system) that does not have cross-reactivity with bovine albumin, rabbit ant-Iba1 antibody (1∶200, WAKO pure chemical industries) and rabbit ant-CD11b antibody (1∶200, Abcam). The cells were rinsed in PBS and incubated for 1 hr at room temperature with tetramethylrhodamine isothiocyanate (TRITC)-conjugated anti-mouse IgG (1∶500, Molecular Probes) and fluorescein isothiocyanate (FITC)-conjugated anti-rabbit IgG (1∶500, Molecular Probes). After wash in PBS, coverslips were mounted onto glass slides using Fluoroguard Antifade reagent (Bio-Rad Laboratories), and examined under a laser confocal fluorescence microscope (FV500, Olympus).

### Immunohistochemistry (IHC)

IHC was performed as previously described [27]. Human fetal (23 wks), adult and Alzheimer's brain tissues were acquired from the Brain bank of Seoul National University hospital. The use of human brain tissues was approved by the institutional review board of Clinical Research Institute, Seoul National University hospital. Briefly, human adult, fetal brain tissues were fixed in 4% paraformaldehyde in 0.1 M phosphate buffer, followed by cryoprotection in 30% sucrose for overnight, and then 30 µm sections were prepared on a cryostat (Leica CM 1900). Paraffin-embedded 4 µm thick Alzheimer's brain tissue sections were de-paraffinized in xylene and rehydrated in a graded ethanol series. Antigen retrieval was performed by immersing slides in citrate buffer (pH 6) at 100°C for 30 min. 10% normal goat serum was used to block non-specific binding. The tissue sections were incubated for overnight at 4°C with mouse anti-albumin (1∶100, R&D system), rabbit anti-Iba1 (1∶200, WAKO), rabbit anti-CD11b (1∶200, Abcam), rabbit anti-GFAP (1∶200, Chemicon), rabbit anti-MAP2 (1∶200, Chemicon) and rabbit anti-MBP (1∶200, Chemicon). The tissues were rinsed in PBS and incubated for 1 hr at room temperature with TRITC-conjugated anti-mouse IgG (1∶500, Molecular Probes) and FITC-conjugated anti-rabbit IgG (1∶500, Molecular Probes). After wash in PBS, coverslips were mounted onto glass slides using Fluoroguard Antifade reagent (Bio-Rad Laboratories), and examined under a laser confocal fluorescence microscope (FV500, Olympus).

### Immunoprecipitation of albumin

Cell lysates were prepared with RIPA buffer containing 1M Tris (pH 7.5), 5M NaCl, 10% NP-40, 10% deoxycholate and protease cocktail inhibitor. 1 mg of cell lysates were incubated with 100 µl of anti-albumin Ab (Abcam)-conjugated Sepharose bead in 500 µl PBS at 4°C for overnight. The Sepharose beads were precipitated at 14,000 rpm for 5 min, and washed with 1 ml of washing buffer containing 50 mM Tris–Cl and 500 mM NaCl (pH 8.0) for three times. The bound complexes were resolved on a 4–12% polyacrylamide gel (Invitrogen), and Coomassie-stained.

### Protein sequencing using tandem mass spectrometry (MS/MS)

Gel bands were excised and subjected to in-gel digestion and MS/MS as described before [28]. All MS/MS experiments were performed using a Nano- LC/MS system consisting of a Surveyor HPLC system and a 7-tesla Finningan LTQ-FT mass spectrometer (Thermo Electron) equipped with a nano-ESI source. For peptide identification, MS/MS spectra were searched using Mascot version 2.0 (Matrix Science). Proteins that were identified by one or more high scoring peptides were considered to be true matches. The high scoring peptides corresponded to peptides that were above the threshold in our Mascot search (expected<0.05, peptide score>28). All high scoring MS/MS spectra were also manually validated.

### Statistical analysis

Results are presented as mean s.e.m. Two-tailed Student *t*-tests were used for data analysis. A p-value of <0.05 was considered as the criteria of statistical significance.

## Supporting Information

Table S1 Albumin peptides identified by MS/MS.
(0.04 MB DOC)
