# Supplementary material for: Human Microglial Cells Synthesize Albumin in Brain
Source: PLoS One. 2008 Jul 30;3(7):e2829. doi: 10.1371/journal.pone.0002829 (PMC2483733; doi:10.1371/journal.pone.0002829)
Supplement: Table S1 — Albumin peptides identified by MS/MS. (0.04 MB DOC) [file pone.0002829.s001.doc]

**Table S1. Albumin peptides identified by MS/MS**

| **Protein** | **Observed** | **Charge** | **Mr (expt)** | **Mr(calc)** | **Delta** | **Peptide**  **ion Score** | **pepide sequence** | **Human**  **specific^*^** |
| --- | --- | --- | --- | --- | --- | --- | --- | --- |
| ALB (IPI00022434) | 409.5405 | 3 | 1225.5996 | 1225.5978 | 0.0017 | 35.55 | FKDLGEENFK | O |
|  | 575.3112 | 2 | 1148.6078 | 1148.6077 | 0.0001 | 62.83 | LVNEVTEFAK | X |
|  | 717.7713 | 2 | 1433.528 | 1433.5261 | 1.0019 | 12.15 | ETYGEMADCCAK | O |
|  | 566.5943 | 3 | 1696.7611 | 1696.7627 | -0.0017 | 32.7 | QEPERNECFLQHK | O |
|  | 464.2504 | 2 | 926.4862 | 926.4861 | 0.0001 | 43.7 | YLYEIAR | X |
|  | 696.285 | 3 | 2085.8333 | 2085.8302 | 0.003 | 49.72 | VHTECCHGDLLECADDR | O |
|  | 725.3246 | 2 | 1442.6379 | 1442.6347 | -0.0001 | 67.89 | YICENQDSISSK | O |
|  | 992.1225 | 3 | 2973.3457 | 2973.3371 | 0.0085 | 63.23 | SHCIAEVENDEMPADLPSLAADFVESK | O |
|  | 820.3983 | 2 | 1638.782 | 1638.7751 | 0.0068 | 67.62 | DVFLGMFLYEYAR | O |
|  | 734.4257 | 2 | 1466.8373 | 1466.8357 | 0.0011 | 74.2 | RHPDYSVVLLLR | O |
|  | 820.8709 | 2 | 1639.7273 | 1639.7188 | 0.0085 | 77.92 | QNCELFEQLGEYK | O |
|  | 480.7849 | 2 | 959.5553 | 959.5552 | 0.0001 | 42.27 | FQNALLVR | O |
|  | 547.3176 | 3 | 1638.9287 | 1638.9304 | 0.0004 | 89.2 | KVPQVSTPTLVEVSR | X |
|  | 756.4253 | 2 | 1510.837 | 1510.8355 | 0.0006 | 76.78 | VPQVSTPTLVEVSR | X |
|  | 500.8057 | 2 | 999.5983 | 999.5964 | 0.0004 | 57.11 | QTALVELVK | X |
|  | 671.8216 | 2 | 1341.6303 | 1341.6274 | 0.0012 | 74.67 | AVMDDFAAFVEK | O |

Mr, molecular weight; expt, expected; calc, calculated.

*Human specific peptides (O) are not 100% homologous with bovine peptides, and thus distinguishable from their bovine homologues using MS/MS. Non-specific peptides (X) have 100% homology with bovine peptides.
